# Large Strain Extrusion Machining of 7075 Aluminum Alloy with Micro-Textured Tools and Analysis of Chip Morphology and Microstructure

**DOI:** 10.3390/mi16121327

**Published:** 2025-11-26

**Authors:** Xiaolong Yin, Minghui Yang, Wan Wang, Youhua Li, Yuying Li

**Affiliations:** 1 School of Mechatronics Engineering, Zhongyuan University of Technology, Zhengzhou 450007, China; 2 Zhengzhou Research Institute of Mechanical Engineering, China Academy of Machinery, Zhengzhou 450001, China

**Keywords:** microtextured tools, large strain extrusion machining, chip morphology, serrated chip, microstructure

## Abstract

Large Strain Extrusion Machining (LSEM) is an intensive plastic deformation process evolved from conventional machining, enabling effective control over chip morphology and grain refinement. This process often generates high cutting temperatures and frictional instability during machining, which degrades material properties and accelerates tool wear. This study proposes a technique combining microtextured tools with LSEM to optimize cutting performance. By designing different microtextured tools (parallel-to-cutting-edge microtextured tools (P-T) and perpendicular-to-cutting-edge microtextured tools (V-T)), cutting experiments were conducted on 7075 aluminum alloy to systematically investigate the effects of microtextured LSEM on cutting performance and chip formation. Results indicate that microtextured tools effectively reduce cutting temperatures. Compared to non-textured tools (N-T), microtextured tools can lower maximum cutting temperatures by up to 13.20% (36.56 °C). Microtextured LSEM suppresses serration formation, leading to more stable chip formation. The serration degree of chips produced by microtextured tools was reduced by up to 25.66% compared to N-T tools. XRD analysis indicates that microtextured tools significantly increase chip dislocation density, reaching nearly 2.77 times that of N-T tools, enhancing material microhardness and refining grain size. This study confirms that combining microtextured tools with LSEM synergistically optimizes chip morphology and improves the microstructural properties of Al7075, providing technical support for machining high-strength aluminum alloys.

## 1. Introduction

Due to their high strength and low density, 7-series aluminum alloys are widely used in mechanical equipment manufacturing, transportation equipment, power system components, and aerospace engineering [1]. Their plastic properties lead to the formation of continuous long chips during machining, which can become entangled with the tool or workpiece. This causes scratches on the machined surface, reducing machining efficiency and safety.

Large Strain Extrusion Machining (LSEM) [2] employs restrictive blocks to macroscopically guide chip deformation pathways, effectively controlling chip morphology and refining material grain structure. However, when material enters the extrusion channel to form chips, cutting temperatures rise sharply. This not only accelerates tool wear and degrades chip material properties but also causes excessive friction at the tool-chip interface to impede material flow. This leads to machining instability and compromises chip morphology control.

Research by Efe et al. [3] indicates that after LSEM treatment, the microstructure of AZ31 magnesium alloy is significantly refined to an ultrafine grain level, with hardness nearly doubling compared to the original material. Sevier et al. [4] found that the compression ratio *λ* dominates strain distribution. A low compression ratio can induce ultra-high strain, significantly refining grains but accompanied by greater extrusion pressure, leading to increased tool-chip friction and making material extrusion more difficult. Deng et al. [5] experimentally and numerically confirmed that reduced compression ratio increases tool-chip friction, elevating equivalent strain and cutting temperature. This hinders material flow and triggers machining instability. Pi et al. [6] identified that cutting heat in extrusion machining primarily originates from material shear deformation and tool-chip friction. Excessive cutting temperatures trigger dynamic recovery effects within the material, adversely affecting its properties. Optimizing machining parameters effectively reduces cutting temperatures, thereby preventing grain growth and material degradation caused by high temperatures. Cai et al. [7] observed that serrated chips cause fluctuating cutting forces, reduced tool life and degraded surface finish during machining. LSEM effectively suppresses serrated chip formation. Wang et al. [8] investigated through finite element analysis that adding microtexture to the tool surface during the LSEM of 7A04 aluminum alloy can enhance the material’s equivalent plastic strain and reduce cutting forces.

Microtextured tools, as a novel surface modification technique, exhibit superior tribological and thermodynamic control capabilities by incorporating micrometer-scale grooves, pits, or other structures on the rake or flank faces [9]. Jahaziel et al. [10] found that microtextured tools cutting thermally conductive metals can reduce peak cutting temperatures by approximately 20% and extend tool life by about 60%. Sun et al. [11] discovered that during dry cutting of titanium alloys, microtextured tools effectively reduce the tool-chip contact area, lowering the friction coefficient by 14%. Sugihara et al. [12] revealed that the texture geometry and dimensions of microtextures determine tool performance, with surface textures altering chip flow patterns to enhance stability. Wang et al. [13] demonstrated that microtextured tools oriented perpendicular to the cutting edge significantly reduce cutting forces and improve surface finish by guiding lubrication and promoting chip evacuation. Currently, most research focuses on conventional cutting. Few studies have explored the introduction of microtexture technology into LSEM and investigated its cutting performance and chip morphology. Research on the mechanisms by which different microtextured tools influence cutting performance, chip morphology and microstructural evolution remains insufficient.

Microtextured tools primarily influence chip flow by acting on the tool-chip contact interface through micro-friction and microtexture geometry [14], whereas LSEM directly intervenes in the overall deformation path of the chip via macro-geometric constraints. The innovative integration of microtextured tools into LSEM processes leverages their friction-reducing and heat-dissipating advantages to directly optimize LSEM parameters, enabling more precise and effective control over chip morphology. Therefore, using 7075 aluminum alloy as the subject, systematically comparing cutting temperatures, chip morphology and microstructural evolution patterns during LSEM using different microtextured tools (parallel-to-cutting-edge microtextured tools (P-T) and perpendicular-to-cutting-edge microtextured tools (V-T)). This provides new insights for overcoming LSEM process bottlenecks and achieving efficient machining of high-performance aluminum alloys.

## 2. Principle of LSEM

**Figure 1:**
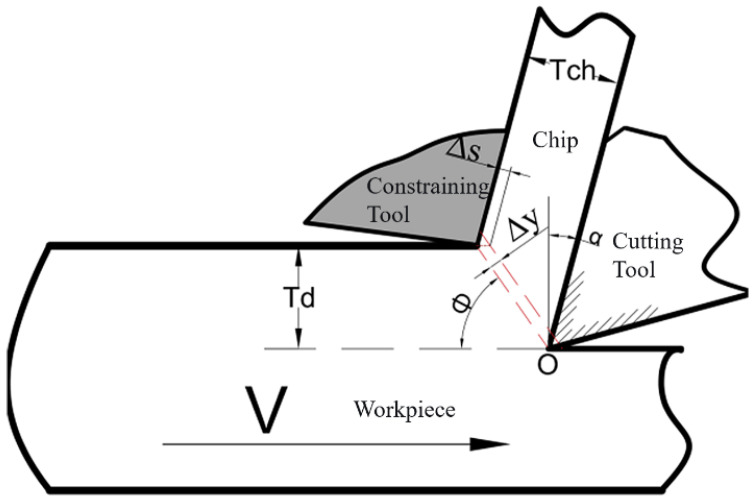
LSEM Schematic Diagram.

LSEM is a strip processing method that combines cutting with extrusion. This technique utilizes an extrusion mechanism to expel chips, yielding nanomaterials with regular geometric shapes, uniform internal structures and high strength [15]. The working principle is illustrated in Figure 1. A restrictive block is added to conventional cutting. Under the combined squeezing force of the cutting tool and the restrictive block, the workpiece is extruded along the channel formed by both components to create chips. During this process, the metal in the cutting layer undergoes severe plastic deformation due to the dual effects of cutting and extrusion.

The resulting shear strain ε [16] is as follows:

(1) $$\varepsilon =\frac{\lambda }{\cos\alpha }+\frac{1}{\lambda \cos\alpha }-2\tan\alpha$$

Strain rate $\dot{\varepsilon }$ [17] is as follows:

(2) $$\begin{array}{c} \dot{\varepsilon }=\frac{\cos\alpha }{\cos\left(\varphi -\alpha \right)}\frac{V}{\Delta y} \end{array}$$

Shear angle φ [18] is as follows:

(3) $$\varphi =\arctan\left(\frac{\cos\alpha }{\lambda -\sin\alpha }\right)$$

In the formula: *a* is the rake angle of the cutting tool; *λ* is the chip thickness compression ratio, defined as the ratio of chip thickness $T_{\mathrm{ch}}$ to cutting layer thickness $T_{d}$ [19], i.e.:

(4) $$\lambda =\frac{T_{\mathrm{ch}}}{T_{d}}$$

## 3. Experiments and Methods

### 3.1. Preparation of Microtextured Tools

**Figure 2:**
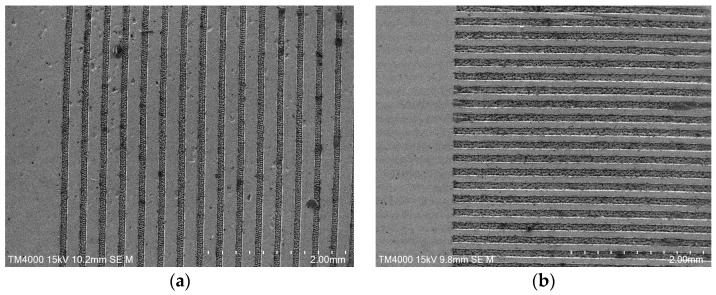
SEM scan of tool front face microtexture: (**a**) P-T tool; (**b**) V-T tool.

This study employs laser processing to create microtextures on tool surfaces. A fiber laser marking machine (YDFLP-20-LP-S model, Dezhong Laser, Yangjiang, China) was used to fabricate microtextures on cemented carbide. Processing parameters were as follows: laser scanning speed 10 mm/s, frequency 800 kHz, scanning number of 5 passes, power setting 63%. Two tool types were produced: P-T tools with texture direction parallel to the cutting edge and V-T tools with texture direction perpendicular to the cutting edge. The texture parameters are as follows: pitch 50 μm, depth 50 μm and width 40 μm. The processed textures were inspected using a profilometer to ensure dimensional compliance with the set texture parameters. The processed texture morphologies are shown in Figure 2.

### 3.2. LSEM Experiment

The experimental material selected was 7075-T6 aluminum alloy tubing with an outer diameter of 60 mm and an inner diameter of 55 mm. Its chemical composition was as follows: 5.6% Zn, 2.50% Mg, 1.6% Cu, 0.5% Fe, 0.40% Si, 0.30% Mn, 0.23% Cr, 0.20% Ti, with the remainder being Al [20]. The cutting tool material is WC-8Co cemented carbide.

**Figure 3:**
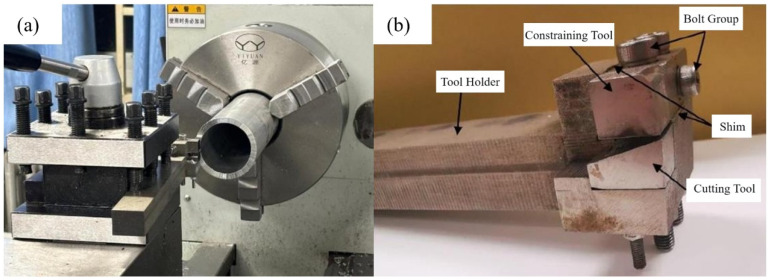
LSEM experimental setup: (**a**) CA6140 lathe (Note: The Chinese warning label on the lathe is translated as “Warning: Be sure to lubricate during use.”); (**b**) combination tool.

The LSEM experiments in this study were conducted on a CA6140 lathe. The workpiece material was clamped in the lathe, while the combined tooling was secured to the lathe tool holder, as shown in Figure 3a. The combined tooling assembly is depicted in Figure 3b. The tool holder, cutting tool and limiting block of this assembly are all bolted connections, allowing for disassembly. This facilitates timely handling of issues such as chip jamming and enables the replacement of cutting tools at any time, thereby altering the type of cutting tool employed. The experimental processing parameters are shown in Table 1.

In the turning experiment, the HIKMICRO-TM series handheld high-frame-rate infrared thermometer from Hikvision was employed. With an emissivity setting of 0.75, it utilized non-contact thermal imaging technology to capture high temperatures during the LSEM process. After the cutting reached a steady state, the thermometer was aimed at the chip discharge point to capture cutting temperatures. The surfaces of the prepared chip specimens were polished to a mirror finish using a metallographic specimen polishing machine (Model MP-2). During polishing, the specimen surface underwent coarse polishing followed by refinement using diamond polishing disks ranging from 800 to 1500 grit. Finally, metallographic polishing compounds from 5.00 μm to 0.25 μm were applied to achieve a mirror finish [21]. The specimen was ultrasonically cleaned for 15 min, and the polished surface was etched using Keller’s reagent solution. Finally, the cross-sectional morphology of the chips was observed using a Leica DFC320 digital metallographic microscope. The longitudinal geometric parameters of the chips were measured and annotated using a super-depth-of-field microscope. The surface hardness of the chips was tested using an automatic turret digital display Vickers hardness tester (Model HVS-30Z, Shanghai Lianer Testing Equipment Co., Ltd.). The load was set at 500 g, held for 10 s, with 10 points tested at different locations on each specimen. The average value was taken as the hardness value. The prepared chips were mechanically ground and electrolytically polished to form test specimens measuring 10 × 10 × 2 mm^3^. X-ray diffraction analysis was performed on each chip using an XRD instrument. The scanning range was set to 20–90° with a step size of 0.02° and a scanning speed of 4°/min for phase analysis of the chips.

## 4. Results and Analysis

### 4.1. Cutting Temperature

Under constant conditions of feed rate *f* = 0.69 mm/r, depth of cut $a_{p}=0.5\mathrm{mm}$ and compression ratio of *λ* = 1.4, real-time temperatures were recorded for three tool types at different cutting speeds. Ten sets of measurements were taken at each speed, and thermal imaging data were analyzed.

**Figure 4:**
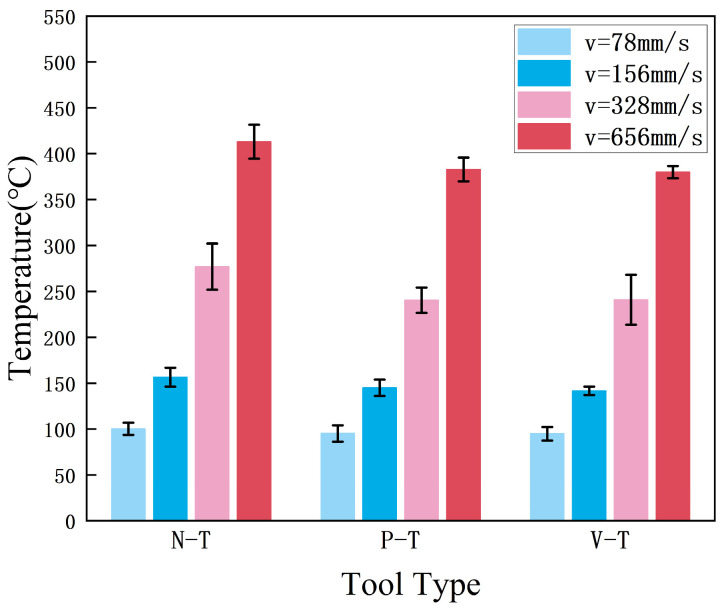
Average maximum cutting temperatures for different tools.

Figure 4 shows the average of the maximum temperature values. The experimental data indicate that cutting temperature is positively correlated with cutting speed, and the temperatures during LSEM with microtextured tools are consistently lower than those with non-textured tools. At *v* = 4.68 m/min and *v* = 9.36 m/min, the average cutting temperatures of all three tools were roughly equivalent. This occurs because at low speeds, the chip flow velocity is low, making aluminum alloy cutting prone to producing continuous ribbon chips. At these speeds, the debris-trapping function of microtexture is suppressed, the interaction pattern between the chip and the textured grooves changes, and the friction coefficient increases which intensifies secondary plowing of the tool surface by the chip. When the workpiece speed increases to *v* = 19.68 m/min and *v* = 39.36 m/min, the average cutting temperatures of both P-T and V-T tools are significantly lower than those of the N-T tool, demonstrating their advantage in reducing cutting temperatures [22]. Compared to N-T tools, P-T tools and V-T tools can reduce the maximum cutting temperature by an average of 8.22% (20.82 °C) and 8.97% (22.71 °C), respectively, with maximum reductions reaching 13.20% (36.56 °C) and 13.02% (36.06 °C), respectively.

Microtextures guide chip flow along specific directions—namely, along the texture orientation—thereby reducing chip adhesion time on the tool surface and minimizing the formation of localized high-temperature zones. Additionally, microtextures enhance heat transfer efficiency to the surrounding environment by increasing the effective heat dissipation area on the tool surface [23].

### 4.2. Chip Morphology

**Figure 5:**
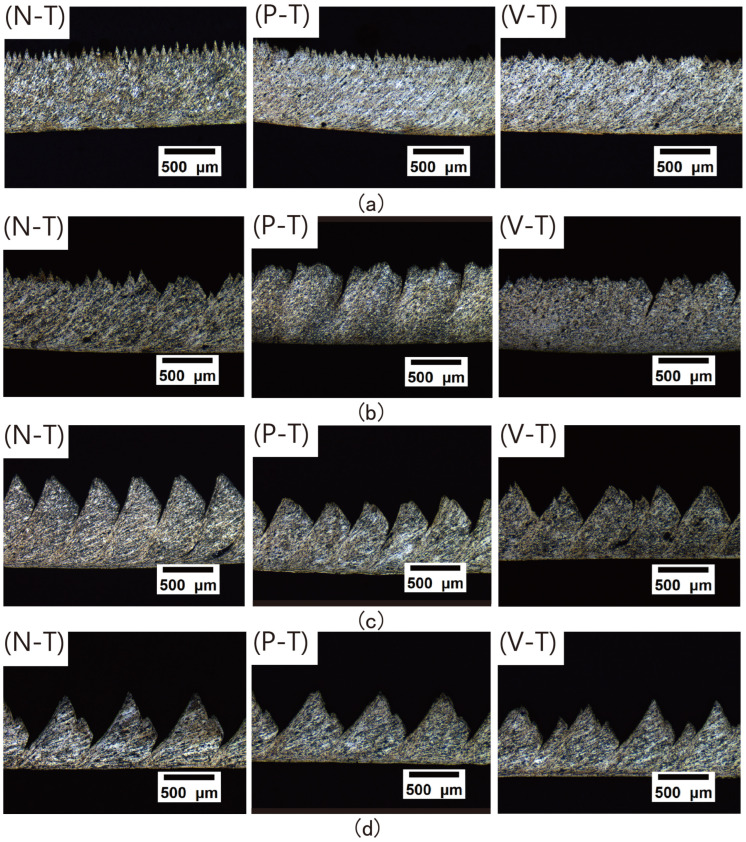
Microscopic images of chip serration: (**a**) *v* = 4.68 m/min; (**b**) *v* = 9.36 m/min; (**c**) *v* = 19.68 m/min; (**d**) *v* = 39.36 m/min.

Under fixed conditions of feed rate (*f* = 0.69 mm/r), depth of cut ( $a_{p\;}=0.5\mathrm{mm}$) and compression ratio of (*λ* = 1.4), the cross-sectional morphology of chips generated by three tool types (N-T, P-T, V-T) during Al7075 machining at different cutting speeds was systematically observed, as shown in Figure 5. The results indicate that at low cutting speeds of *v* = 4.68 m/min and *v* = 9.36 m/min, the chips produced by all three tools exhibited no distinct serrated features, instead forming continuous ribbon-like structures with wavy patterns.

This stems from uniform material deformation, lower temperatures in the shear zone and stable plastic flow, resulting in continuous ribbon-like chips. As cutting speed increases, serration gradually intensifies [24]. Comparing the serration phenomena among the three tools, the N-T tool exhibited more pronounced serrations than the P-T and V-T tools. This occurs because increased cutting speed elevates strain and strain rate, intensifying deformation. Concentrated shear slip then causes a sharp rise in cutting temperature, leading to more evident chip serration [25]. Combined with the aforementioned ability of microtextured tools to reduce cutting temperatures, this indicates that microtextured tools can suppress the formation of serrations in chips.

**Figure 6:**
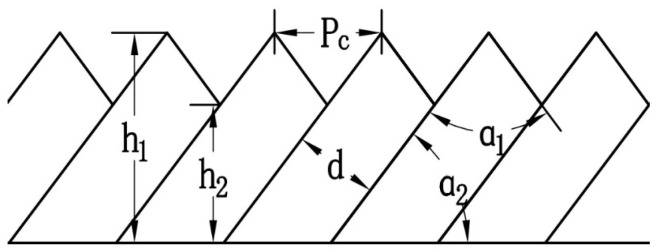
Chip Serration Parameters Diagram.

Adiabatic Shear Theory [26] posits that during chip formation, workpiece material undergoes plastic deformation due to compression. When this plastic deformation causes localized temperature increases in the workpiece material, leading to thermal softening exceeding the material’s strain hardening, adiabatic shear occurs. Under the alternating effects of thermal softening and strain hardening, periodic shear bands [27] form in the material. Parameters describing the serration morphology include serration degree $G_{s}$, serration pitch $P_{c}$, adiabatic shear band spacing *d*, rake angle $\alpha_{1}$ and clearance angle $\alpha_{2}$, as shown in Figure 6. The degree of serration in serrated chips can be expressed in two forms. We adopt the serration degree $G_{s}$ definition proposed by Schulz H. et al. [28], calculated as follows:

(5) $$G_{s}=\frac{h_{1}-h_{2}}{h_{1}}$$

where $h_{1}$ is the tooth crest height, $h_{2}$ is the tooth root height and a larger $G_{s}$ value indicates a more severe degree of serration in the serrated chips.

Due to the low yield strength of Al7075 (approximately 500 MPa) and its uniform plastic deformation, the serration degree is relatively small, resulting in less pronounced serration during low-speed cutting. Therefore, chips from two speed groups—*v* = 19.68 m/min and *v* = 39.36 m/min—were selected for measurement. The serration degree $G_{s}$ and tooth pitch $P_{c}$ were statistically analyzed and averaged.

**Figure 7:**
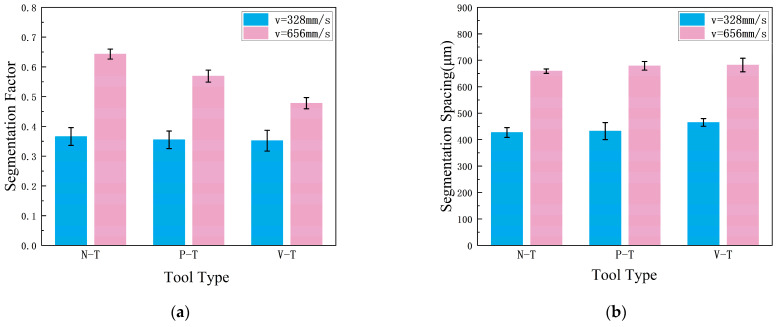
Chip serration: (**a**) Serration degree $G_{s}$; (**b**) Tooth-peak spacing $P_{c}$.

Figure 7 shows the variation in chip serration with cutting speed. Comparison reveals that under identical speed conditions, chips produced by both microtextured tools exhibit lower serration than those from conventional tools at the same speed. At both cutting speeds, the serration values for the N-T tool chips were 0.366 and 0.643, while microtextured tool chips showed values of 0.355 and 0.569 for P-T chips and 0.352 and 0.478 for V-T chips. Both microtextured tools produced chips with lower serration than the non-textured tool. Compared to the N-T tool, the P-T chip serration decreased by 3.01% and 11.51%, while the V-T chip serration decreased by 3.83% and 25.66%. As shown in Figure 7a, the serration degree of chips gradually increases with rising cutting speed. Higher speeds generate more friction heat, intensify extrusion deformation and exacerbate shear slippage, resulting in more pronounced serration patterns. Figure 7b presents the statistical distribution of chip serration pitch ( $P_{c}$). It is evident that the serration pitch of chips produced by all three tools gradually increases with cutting speed. Higher cutting speeds elevate strain rate and local temperature, intensifying adiabatic shear localization and consequently widening the serration pitch. Furthermore, comparing microtextured tools with conventional tools reveals that the serration spacing is larger for microtextured tools. This is because microtextured tools reduce the contact area with the chip during contact, lowering temperature, decreasing plastic deformation and relatively weakening slip phenomena, thereby suppressing serrations [29]. Comparing tooth spacing values at different cutting speeds reveals that the difference between microtextured and conventional tools is greatest at 39.36 m/min. This indicates that microtexturing more effectively suppresses tooth formation at this speed, thereby improving chip formation. Comparing two different microtextured tools, the V-T tool’s texture direction aligns with the chip flow direction, facilitating smooth chip evacuation. The P-T tool’s texture direction is perpendicular to the chip flow direction, causing secondary cutting and resulting in relatively higher cutting temperatures compared to the V-T tool. Higher cutting temperatures intensify shear slippage and increase chip serration. Consequently, V-T tools demonstrate superior performance in suppressing chip serration.

### 4.3. Microstructure of Chips

**Figure 8:**
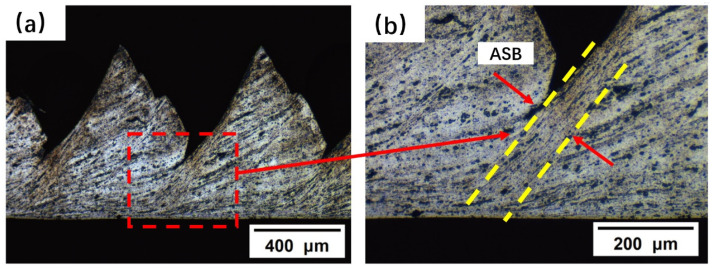
(**a**) Metallographic microstructure of serrated chip morphology; (**b**) Metallographic microstructure of the shear band (ASB).

During low-speed cutting, work hardening dominates the thermal deformation behavior of the material. As the cutting speed gradually increases, the local temperature in the first deformation zone rises significantly. When adiabatic shearing occurs, forming periodic shear bands, it triggers dynamic recrystallization (DRX) [30]. With further increases in temperature and strain rate, the process transitions from continuous dynamic recrystallization to geometric dynamic recrystallization [31]. Figure 8 shows the chip morphology observed under a metallurgical microscope at a cutting speed of *v* = 39.36 m/min. Analysis indicates that the serrated chips exhibit a typical periodic adiabatic shear band structure. The shear band width ranges from approximately 60 to 90 μm. Significant grain refinement is observed within the shear bands, where primary grains are elongated into fibrous structures along the shear direction. Localized regions exhibit ultrafine equiaxed grains (<2 μm) formed by dynamic recrystallization.

**Figure 9:**
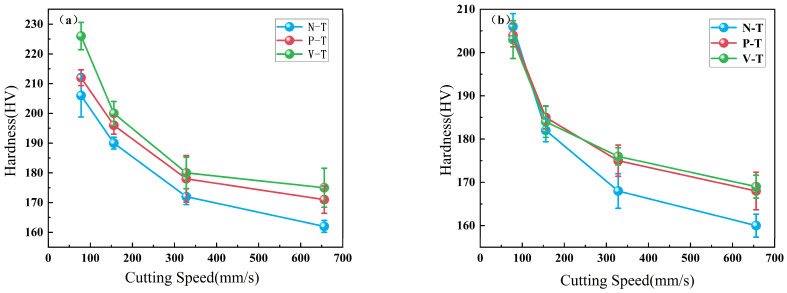
(**a**): Hardness of shear slip zone at different cutting speeds; (**b**): Hardness of matrix zone at different cutting speeds.

Comparing the hardness of the chip matrix zone and shear slip zone produced by three different tools, experimental data demonstrate that varying degrees of plastic deformation across different regions result in differing chip hardness. Organizing the measured results, the line graph in Figure 9 shows that for the same tool under identical feed rate and depth of cut conditions, chip hardness gradually decreases with increasing cutting speed. The rate of hardness reduction also diminishes as cutting speeds increases, attributed to the narrowing gap between material strain hardening and thermal softening effects. Comparing identical parameters, the hardness in the shear slip zone consistently exceeds that of the matrix region. This is attributed to severe compression slip in the shear slip zone, which enhances hardness [32]. The shear zone exhibits a hardness 10–20% higher than the matrix. Chips produced by microtextured tools generally demonstrate higher hardness than those from conventional tools.

**Figure 10:**
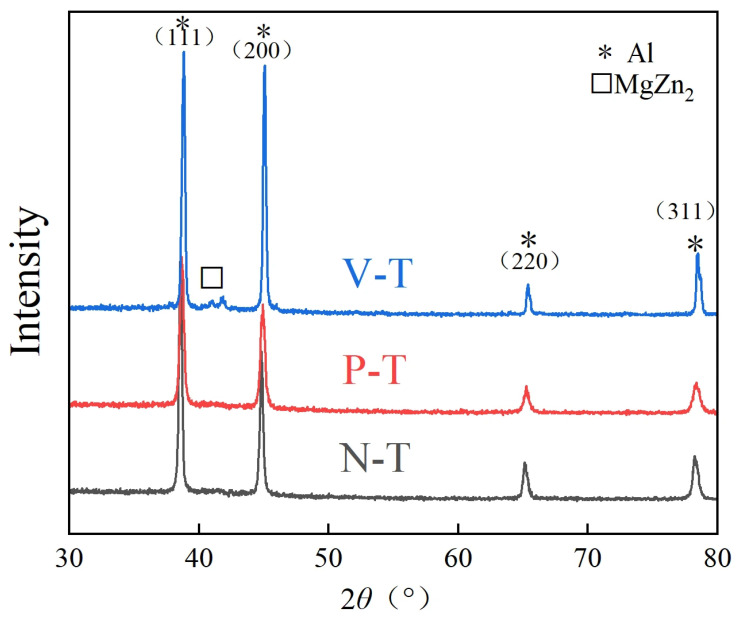
XRD phase analysis of chip specimens.

The results of the XRD phase analysis for chips produced by microtextured and conventional non-textured tools at a cutting speed of 39.36 m/min are shown in Figure 10.

Al7075 belongs to the Al-Zn-Mg-Cu series of alloys, with the precipitation sequence being as follows: supersaturated solid solution—GP zone—*η*′(MgZn)—*η*(MgZn_2_). Figure 10 shows a small peak near 2*θ* = 42° in the N-T tool chip sample, corresponding to the MgZn_2_ precipitation phase—the *η*’ phase transformed from the GP region. This peak is very weak in the microtextured tool (P-T and V-T) chip samples. XRD phase analysis indicates that the volume fraction of *η*’ phase (MgZn_2_) in chips decreases with microtextured tools compared to the N-T tool. Four characteristic diffraction peaks were identified: (111), (200), (220) and (311) corresponding to α-Al crystal planes. Comparing the three sets of XRD patterns, the angular shifts in the main Al phase diffraction peaks (111, 200, 220) among the three tools were minimal, indicating insignificant lattice constant changes and negligible residual stress differences. Chips produced by the three tools exhibit regular fluctuations in peak intensity. Due to preferential orientation during hot extrusion deformation, the intensity of the (200) crystal plane diffraction peak varies, showing both strong and weak signals. The groove texture grooves on microtextured tools induce multidirectional slip during chip sliding, thereby reducing the (111) peak intensity. Microtexture may cause anisotropy in the mechanical properties of chips, while the random orientation of microtextured tools helps improve material uniformity.

Under LSEM processes, the core microstructural features within metallic materials manifest as high-density dislocation networks. The proliferation and evolution of dislocations. Precise characterization of dislocation density and the establishment of computational models are crucial for analyzing microstructural evolution patterns and quantitatively assessing dislocation strengthening mechanisms [33]. Based on crystal plasticity theory, the dislocation density in materials after extrusion and cutting can be calculated using the following equation [34,35]:

(6) $$\rho =\frac{2\sqrt{3}{\langle \varepsilon^{2}\rangle }^{\frac{1}{2}}}{\left(d_{\mathrm{XRD}}*b\right)}$$

where b is the Burgers vector with a value of 0.286 nm; ${\langle \varepsilon^{2}\rangle }^{1/2}$ represents the material’s microstrain; $d_{\mathrm{XRD}}$ denotes the grain size. These parameters can be calculated using the full width at half maximum (FWHM) of the XRD diffraction peak [36]. FWHM *β* consists of two components: grain size-induced broadening *β*_1_ and microstrain-induced broadening *β*_2_. These can be estimated using the Cauchy and Gaussian equations, respectively. Combining Scherrer and Wilson’s methods yields the following formula [37]:

(7) $$\begin{array}{c} \frac{\beta^{2}}{\tan^{2}\theta }=\frac{\lambda_{XRD}}{d_{\mathrm{XRD}}}\left(\frac{\beta }{\tan\theta \sin\theta }\right)+16\varepsilon^{2} \end{array}$$

where,

β: Half-width at half maximum of the diffraction peak;

θ: Half-diffraction angle;

$\lambda_{XRD}$: X-ray wavelength, $\lambda_{XRD}$ = 0.15405 nm.

**Figure 11:**
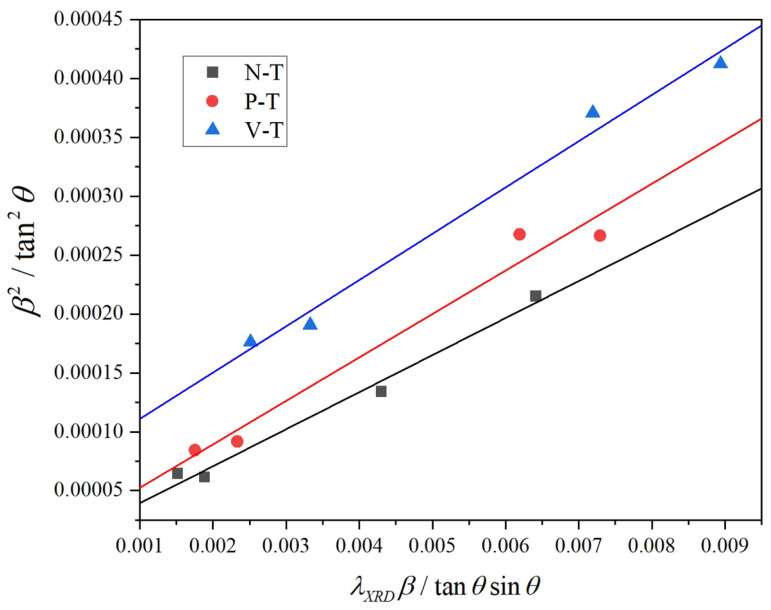
XRD Diffraction Data Fitting.

Measurements of the positions and half-widths of the four strongest peaks (111), (200), (220) and (311) in the chip specimens produced by N-T, P-T and V-T tools were conducted. Let $\frac{\beta^{2}}{\tan^{2}\theta }$ represent *y* in the binary linear equation, $\lambda_{XRD}\left(\frac{\beta }{\tan\theta \sin\theta }\right)$ as *x*, with the slope of the line being 1/ $d_{\mathrm{XRD}}$ and the intercept being 16 $\varepsilon^{2}$. Substituting the measured values into the equation yields $d_{\mathrm{XRD}}$ and ε, with specific data shown in Table 2 and Figure 11. Substituting these values into the formula, the dislocation densities for N-T, P-T and V-T chip specimens are determined to be 2.68 × 10^14^ m^−2^, 4.42 × 10^14^ m^−2^, and 1.01 × 10^15^ m^−2^, respectively. Compared to N-T tools, microtextured tools produce smaller grain sizes during cutting. This is because microtextured tools effectively reduce the friction coefficient in the tool-chip contact zone, thereby minimizing heat accumulation during cutting. Additionally, the lubricating effect of microtexturing reduces cutting force fluctuations and decreases grain elongation deformation [38]. The texture orientation of V-T tools aligns parallel to the chip flow direction. This texture effectively guides chips to flow along the texture direction during cutting, thereby exhibiting superior cooling and friction-reducing effects compared to P-T and N-T tools. At lower cutting temperatures, The dynamic recovery process within the material is suppressed, leading to the accumulation of a large number of dislocations and a significant increase in dislocation density. In contrast, P-T tools operate at higher cutting temperatures than V-T tools, inducing stronger recrystallization. This recrystallization causes grain growth, thereby reducing dislocation density. Consequently, the dislocation density in chips produced by P-T tools is lower than that in chips produced by V-T tools. In future work, we will employ more direct tribological measurement methods to comprehensively demonstrate the optimization mechanism of microtextured tools in LSEM.

## 5. Conclusions

1. Microtextured cutting tools effectively reduce cutting temperatures. Compared to N-T tools, P-T tools and V-T tools reduced maximum cutting temperatures by 8.22% (20.82 °C) and 8.97% (22.71 °C) on average, with maximum reductions of 13.20% (36.56 °C) and 13.02% (36.06 °C), respectively. Microtextures guide chip flow directionality, reducing adhesion time at the tool-chip interface while increasing the tool’s effective heat dissipation area. This enhances heat dissipation efficiency, thereby lowering cutting temperatures and reducing tool wear.
2. Microtextured cutting tools suppress serrated chip formation. Under identical cutting conditions, chips produced by P-T and V-T tools exhibited lower serration ( $G_{s}$) than those from N-T tools (with maximum reductions of 11.51% and 25.66%, respectively). The increased serration spacing enhances chip formation stability, thereby improving machined surface quality.
3. Microtextured cutting tools can enhance the microstructural properties of materials. The microhardness of both shear slip zones and matrix regions in chips produced by the two microtextured tools is generally higher than that in chips produced by the N-T tool. The dislocation density in chips produced by V-T tools (1.01 × 10^15^ m^−2^) was significantly higher than that in chips from P-T tools (4.42 × 10^14^ m^−2^) and N-T tools (2.68 × 10^14^ m^−2^), indicating the potential of microtextured tools for optimizing cutting processes and enhancing material microstructural properties.
4. Comparing the two microtexture tools, the V-T tool demonstrates superior performance, exhibiting more pronounced effects in reducing cutting temperatures, suppressing chip serration and enhancing material micro-strength.
5. Microtextured tools can optimize the LSEM process. This study confirms that introducing microtextured tools into the LSEM process effectively overcomes challenges such as high cutting temperatures and significant tool-chip friction. Microtexturing optimizes the LSEM process of Al7075 across multiple levels—tribology, thermodynamics, and microstructural evolution—by reducing friction, promoting heat dissipation, guiding chip flow, suppressing serration formation, refining grain size, and increasing dislocation density. Provides technical support for the processing of high-strength aluminum alloys.

## Figures and Tables

**Table 1:** Machining parameters for cutting experiment.

Machining Parameter	Value
*v*/(m/min)	4.68, 9.36, 19.68, 39.36
$a_{p}$/(mm)	0.5
*f*/(mm/r)	0.69
*λ*	1.4
$\gamma_{0}$/(°)	10°
$\alpha_{0}$/(°)	5°

**Table 2:** XRD measurement data and calculation results.

Group	2θ (Deg)	Full Width at Half Maximum (FWHM) (Deg)	Microstress Deformation (%)	Grain Size Dimension (nm)	Dislocation Density (m^−2^)
N-T	38.81581	0.29614	0.202	37.3	2.68 × 10^14^
	45.06850	0.27525			
	65.40259	0.28876			
	78.51829	0.37652			
P-T	38.81581	0.32271	0.258	27.8	4.42 × 10^14^
	45.06850	0.38166			
	65.40259	0.34757			
	78.51829	0.42503			
V-T	38.58053	0.40723	0.168	25.6	1.01 × 10^15^
	44.83276	0.45517			
	65.17355	0.50584			
	78.29915	0.61913			

## Data Availability

The raw data supporting the conclusions of this article will be made available by the authors on request.

## Nomenclature

Symbol	Description
$a_{p}$	Depth of cut
ASB	Adiabatic Shear Band
b	The Burgers vector
*d*	Adiabatic shear band spacing
$d_{\mathrm{XRD}}$	The grain size
DRX	Dynamic recrystallization
*f*	Feed rate
$G_{s}$	Serration degree
$h_{1}$	Tooth crest height
$h_{2}$	Tooth root height
LSEM	Large Strain Extrusion Machining
N-T	Non-textured tools
$P_{c}$	Serration pitch
P-T	Parallel-to-cutting-edge microtextured tools
$T_{\mathrm{ch}}$	Chip thickness
$T_{d}$	Cutting layer thickness
V-T	Perpendicular-to-cutting-edge microtextured tools
*V*/*v*	Tool velocity
$\alpha_{0}$	Clearance Angle
$\alpha_{1}$	Rake angle of serrated chip
$\alpha_{2}$	Clearance angle of serrated chip
*α*/ $\gamma_{0}$	Tool rake angle
*β*	Half-width at half maximum of the diffraction peak
*∆y*	Width of the Primary Shear Zone
*ε*	Effective strain
$\dot{\varepsilon }$	Strain rate
*λ*	The chip thickness compression ratio
$\lambda_{\mathrm{XRD}}$	X-ray wavelength
*ρ*	Dislocation density
*φ*	Shear angle
*θ*	Half-diffraction angle
